# Functional conservation of an ancestral Pellino protein in helminth species

**DOI:** 10.1038/srep11687

**Published:** 2015-06-29

**Authors:** Christopher D. Cluxton, Brian E. Caffrey, Gemma K. Kinsella, Paul N. Moynagh, Mario A. Fares, Padraic G. Fallon

**Affiliations:** 1 School of Medicine, Trinity Biomedical Sciences Institute, Trinity College Dublin, Dublin 2, Ireland; 2 Department of Genetics, Trinity College Dublin, Dublin 2, Ireland; 3 Institute of Immunology, National University of Ireland Maynooth, County Kildare, Ireland; 4 Integrative Systems Biology Group, Instituto de Biología Molecular y Celular de Plantas (C.S.I.C-UPV); 5 National Children’s Research Centre, Our Lady’s Children’s Hospital, Dublin 8, Ireland

## Abstract

The immune system of *H. sapiens* has innate signaling pathways that arose in ancestral species. This is exemplified by the discovery of the Toll-like receptor (TLR) pathway using free-living model organisms such as *Drosophila melanogaster*. The TLR pathway is ubiquitous and controls sensitivity to pathogen-associated molecular patterns (PAMPs) in eukaryotes. There is, however, a marked absence of this pathway from the plathyhelminthes, with the exception of the Pellino protein family, which is present in a number of species from this phylum. Helminth Pellino proteins are conserved having high similarity, both at the sequence and predicted structural protein level, with that of human Pellino proteins. Pellino from a model helminth, *Schistosoma mansoni* Pellino (SmPellino), was shown to bind and poly-ubiquitinate human IRAK-1, displaying E3 ligase activity consistent with its human counterparts. When transfected into human cells SmPellino is functional, interacting with signaling proteins and modulating mammalian signaling pathways. Strict conservation of a protein family in species lacking its niche signalling pathway is rare and provides a platform to examine the ancestral functions of Pellino proteins that may translate into novel mechanisms of immune regulation in humans.

The phylum Platyhelminthes (flatworms) consists of approximately 50,000 different species that populate a remarkable variety of niches. In addition to free-living forms, including *Dugesia japonicum* and *Schmidtea mediterranea*, it encompasses parasitic organisms, such as *Clonorchis sinensis*, *Echinococcus granulosus*, *Hymenolepis microstoma*, *Schistosoma japonicum* and *Schistosoma mansoni*, responsible for inflicting debilitating diseases upon hundreds of millions of people throughout the world. Platyhelminthes are considered by many to occupy an important position in the evolution of the Metazoa^1^,^2^ and as such are interesting model organisms to dissect ancestral signalling mechanisms of conserved proteins. Yet, as important, abundant and diverse as platyhelminthes are, little is known about the molecular events that guide their sophisticated and often plastic biological properties. The recent advent of genomic sequencing and the compilation of searchable databases of numerous plathyhelminth species has provided a platform to examine the evolution of plathyhelminthes relative to mammals and indeed, molecular conservation.

Toll-like receptors (TLRs) were originally described in research on *Drosophila*^3^, with the TLR signaling pathway being highly conserved in flies, nematodes, plants and man^4^,^5^. The use of free-living model organisms, such as *D. melanogaster* or *Caenorhabditis elegans*, has facilitated analysis of homologous innate immune signaling pathways in *Homo sapiens*. Upon ligand binding, all TLRs except TLR3 recruit the adaptor protein Myeloid differentiation primary response 88 (MyD88) and the kinases Interleukin-1 receptor-associated kinase (IRAK)-1 and IRAK-4^6^. TLR2 and -4 signalling require the adaptor Myelin and lymphocyte protein (MAL) to bridge the receptor and MyD88^7^. IRAK-4 phosphorylates IRAK-1, leading to IRAK-1 autophosphorylatio n^8^. The kinases then leave the receptor to interact with TNF receptor-associated factor (TRAF)-6, which promotes the generation of unanchored lysine 63 polyubiquitin chains^9^, leading to activation of the downstream kinase Mitogen-activated protein kinase (MAPK) kinase 7 (TAK-1)^10^. This in turn can lead to activation of MAPK signalling as well as stimulation of Inhibitor of kappaB (IκB) kinase (IKK) activity. IKKβ phosphorylates IκB proteins, leading to their ultimate degradation and the ensuing liberation of Nuclear factor (NF)-κB^11^.

An emerging aspect of regulation of TLR signalling is the role of the Pellino family of proteins^12^,^13^. Pellino was first identified in *Drosophila* as a binding partner of Pelle, a *Drosophila* homolog of IRAK^14^. Three members of the mammalian Pellino family have been shown to function as E3 ubiquitin ligases, catalysing K63-linked polyubiquitination of IRAK-1^13^,^15^,^16^. Indeed there exists a bidirectional communication in the IRAK–Pellino associations, in that IRAK-1 and IRAK-4 can phosphorylate Pellino proteins on various serine and threonine residues, thus enhancing the E3 ubiquitin ligase activity of the Pellinos. The latter can then catalyse polyubiquitination of IRAK-1^15^,^17^. The C-terminal regions of the Pellino proteins contain a conserved RING-like domain that confers E3 ubiquitin ligase activity (Fig. 1). Furthermore, a cryptic forkhead-associated (FHA) phosphothreonine-binding module has been identified in the N-terminal region of the protein^18^, which facilitates Pellino interaction with phosphorylated IRAK-1 (Fig. 1). The FHA domain in the Pellino family contains an additional appendage or “wing” that contains multiple IRAK phosphorylation sites^17^.

Here, we examine the molecular conservation of the Pellino protein family in platyhelminthes by a comparative analysis with known human Pellino proteins, and by using Pellino from the parasitic model organisms *S. mansoni*, we identify a functional homologue of a TLR signalling protein conserved in a TLR deficient organism.

## Results

### Characterizing TLR signaling in helminths

The TLR pathway regulates the first line of defense against invading pathogens and plays a significant role in inflammation, immune cell regulation, survival, and proliferation. It is considered ubiquitous having been identified in most organisms from mammals to plants^19^. Their ubiquitous nature makes it possible to identify and functionally characterize mechanisms of signaling in mammals using model organisms. TLRs and the downstream transcription factor NFκB have been previously described as absent from the genomes of platyhelminths, including *S. mansoni*^20^. To determine the conservation of intracellular TLR signaling molecules in platyhelminths, we have formally addressed the ubiquitous nature of the classical pathway proteins (TLR, MyD88, IRAK, TRAF, Pellino, IKK, IκB and NFκB). BLASTp analysis using *Homo sapiens* and *Drosophila melanogaster* query sequences was performed to identify homologues in free-living helminths (*Dugesia japonicum* and *Schmidtea mediterranea)* and parasitic helminthes *(Clonorchis sinensis*, *Echinococcus granulosus*, *Hymenolepis microstoma*, *Schistosoma japonicum* and *S. mansoni*), using a free-living nematode *(Caenorhadbitis elegans)* and parasitic nematode *(Brugia malayi)* as controls (Table 1). TLR homologues were identified only for human, *D. melanogaster* and *C. elegans,* while all other nematode and platyhelminth species were TLR deficient. In fact, all worm species had lost the TLR pathway with the exception of two molecules, TRAF and Pellino (Table 1). While TRAF homologues were identified in all helminths tested, they are more similar to TRAF2, a member of the TNF not TLR signalling pathway. The Pellino protein family is conserved in the helminth and nematode species, however (Table 1). Interestingly, the non-parasitic helminth species did not have a Pellino protein annotated in their databases. There have been Pellino-like EST sequences identified in the genome of *S. mediterranea*, but not *D. japonicum,* however a Pellino protein has yet to be confirmed.

### Homologous Pellino protein sequences from helminths

To formally address the putative helminth Pellino proteins identified from the databases, the sequences were examined *in silico* for characteristic of the Pellino protein family. A functional Pellino motif (InterProScan: IPR006800) was found for each protein tested, having the gene ontology classification GO: 0008063 assigned to the Pellino protein motif. Multiple sequence alignment quantified sequence identity to human Pellino proteins as ~40% ± 5% (see Supplementary Table S1). Interestingly, the truncated N-terminal *S. japonicum* Pellino protein had the same level of sequence similarity with all three human Pellino proteins (see Supplementary Table S1) suggesting strong conservation of the N–terminal region of Pellino proteins with non-conserved regions concentrated in the C-terminal region.

### Helminth Pellino proteins conform to natural speciation patterns

Having identified Pellino proteins in helminths as potentially novel signalling homologues of human Pellino proteins, we performed a comprehensive analysis of their phylogenetic relationship. A phylogenetic tree was constructed from the complete sequence alignment of confirmed Pellino proteins from all available species (including paralogues). To the alignment we added putative protein sequences recently identified in helminth genomes including *Clonorchis sinensis, Hymenolepis microstoma, Echinococcus granulosus* and *Schistosoma japonicum* and *Schistosoma mansoni.* An analysis of the full-length helminth Pellino proteins with its orthologues revealed that the flatworm Pellino proteins are placed as ancestral to the mammal clade and the nematode clade (Fig. 2). The phylogeny of helminth Pellino proteins therefore conforms to natural speciation patterns.

### Homology modelling of helminth Pellino FHA domains

To support the presence of functional helminth FHA domains, comparative models of helminth Pellino proteins were calculated using the amino acid sequences of the two available crystal structures of the HsPellino2 protein from man (PDB: 3EGA at 1.8 Å and PDb: 3egb at 3.3 Å) as templates^18^. Multiple models were generated from both available structures to determine the stability of the helminth Pellino FHA domain (Fig. 3 and supplementary tables 2–4). Following this process, a stable 11-stranded β-sandwich and peripheral β-strand structures remained for helminth Pellinos, for a total 17-stranded structure (see Supplementary Fig. S1, S2 and Supplementary Table S5). A topology-based comparison of our model, SmPellino, with HsPellino2 demonstrates that the β-sandwich has the same strand orientation as that observed for the core FHA domain of HsPellino2 and the peripheral structures constitute the ‘wing’ appendages (Fig. 3). SmPellino and HsPellino2 display almost identical domains, in both strand length and orientation, with the only deviation found in inter-strand length and extended loop regions (Fig. 3). SmPellino, therefore, encodes a core FHA domain, which includes the ‘wing’ appendage that decorates the FHA domain of Pellino proteins.

### Conservation of functional residues of the FHA domain

Given the structural homology of the protein, we next addressed the conservation of known functional residues in SmPellino. The YGEL sequence in human Pellino proteins is important for activation of p38 MAPK^14^ and is conserved in both schistosomes, with *C. sinensis* poorly conserved, and *E. granulosus* and *H. microstoma* containing a Glutamic acid (E) to Arginine (R) substitution (See Supplemental Fig. 3). Different helminths may have p38-independent signalling mechanism or a different interface for protein interaction. Mammalian Pellino proteins can be activated by phosphorylation *in vitro*, catalysed by IRAK1 or IRAK4. The functionality of Pellino proteins has been enhanced by alternative phosphorylation of seven critical amino acid residues. Specifically, it was shown that full activation of Pellino1 can be achieved by phosphorylating any one of several different sites (Ser-76, Thr-86, Thr-288, or Ser-293) or a combination of other sites (Ser-78, Thr-80, and Ser-82)^17^. Residues corresponding to Ser-78, Thr-80 and Thr-288 are conserved in all helminth species, while conservation of other phosphorylation sites is heterogeneous (See Supplemental Fig. 3). There are, however, sufficient sites present on each protein to confer efficient protein activation. Furthermore, helminth Pellino proteins have maintained five highly conserved signature residues characteristic of HsPellino proteins, that are essential for FHA binding to phosphothreonine sites on target proteins R106, S137, R138, T187 and N188 (See Supplemental Fig. 3). Helminth Pellino proteins, therefore, have the capacity to bind phosphothreonine residues on target protein and peptides. This, in conjunction with the homology modeling described above, provides strong predictive indication that SmPellino contains a FHA domain.

### Conservation of the RING-like domain

**Figure 1:**
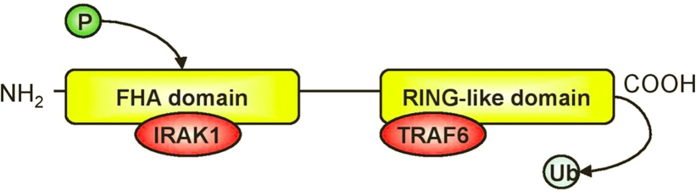
Schematic of Pellino proteins. Pellino proteins contain an N-terminal FHA domain and a C-terminal RING-like domain. The FHA domain contains residues for activation and IRAK binding, while the C-terminal RING-like domain confers E3-ligase activity of the protein. TRAF proteins potentially bind the RING-like domain, as RING domains are known to bind each other *in vivo*.

Mammalian Pellino proteins are efficient E3 ubiquitin ligases, a property of their C-terminal RING-like domain, in which cysteine and histidine residues are arranged in an atypical CHC2CHC2 formation^12^. The RING-like domain of Pellino proteins (Fig. 1) is a feature of all helminth Pellino proteins^21^. Multiple sequence alignment of the C-terminal regions of Pellino sequences with that of helminth Pellinos shows that the helminth protein contains an identical RING-like conformation (Fig. 4).

### Functional conservation of *S. mansoni* Pellino

Having characterized helminth Pellino proteins as having moderate levels of similarity with human Pellinos, while maintaining sequential and structural conformation of functional domains, we characterize the *S. mansoni* Pellino protein, as a model helminth protein, in a mammalian system to examine functional conservation. This approach provides a means to assess the degree to which structural and sequential similarities and differences translate into functional properties in the same system.

In *H. sapiens*, Pellino proteins bind IRAK-1 proteins (Fig. 1), *via* the five conserved phosphothreonine binding residues in the FHA domain^17^, which are present in the parasite homolog (See Supplemental Fig. 3). Human embryonic kidney (HEK)-293 cells were co-transfected with affinity-tagged SmPellino and HsIRAK-1. SmPellino was shown to co-precipitate with HsIRAK-1 (Fig. 5A), as reported with HsPellinos and HsIRAK-1^22^. Furthermore, SmPellino also bound HsTRAF-6 (Fig. 5A), another target protein of HsPellino proteins^23^. The capacity of SmPellino to interact *in vivo* with human target proteins confirms the homology modelling data and supports the prediction that SmPellino contains a fully functional FHA domain (Figs 3 and 5).

**Figure 2:**
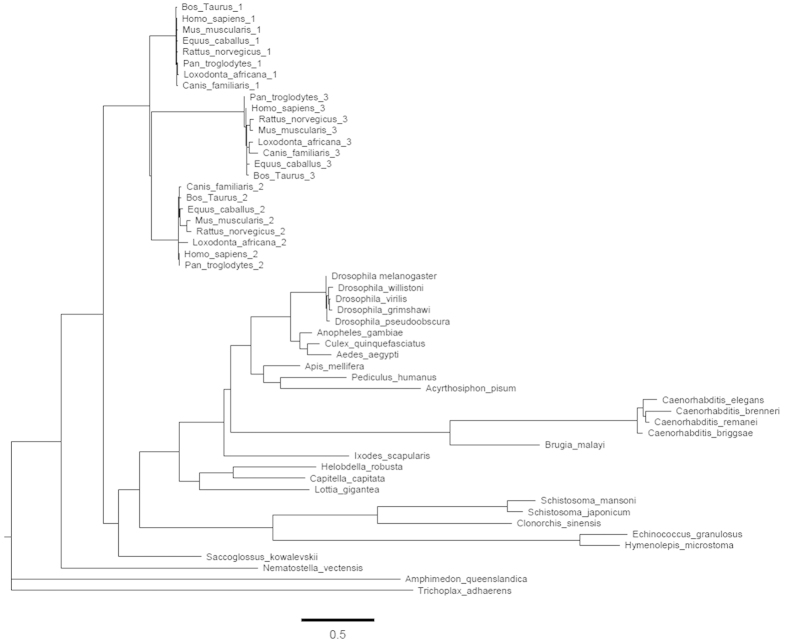
Phylogenetic analysis of the full-length sequences of known mammalian, nematode and arthropod Pellinos with helminth Pellino orthologues. A maximum likelihood tree was formulated using the LG model and gamma correction. 100 bootstraps were calculated. The scale bar represents mutations per site.

The RING-like domain of human Pellinos is essential to manifest E3-ligase activity (Fig. 4)^13^. As shown above (Fig. 4), the SmPellino sequence has an identical RING-domain, with a CHC2CHC2 cysteine and histidine formation, as found in other Pellino sequences, demonstrating that the Pellino family RING-like domain sequence is conserved in SmPellino. As SmPellino can bind HsIRAK-1 (Fig. 5A) we addressed if SmPellino could induce poly-ubiquitination of HsIRAK-1. When co-expressed in HEK 293 cells SmPellino caused marked poly-ubiquitination of HsIRAK-1 (Fig. 5B). These data demonstrate that SmPellino functions as an E3 ubiquitin ligase (Fig. 2).

As there is functional conservation of the protein domains of SmPellino, we next investigated whether SmPellino could modulate mammalian signaling in human cells. This assesses the predicted capacity of the protein to modulate NF-κB signalling in line with its structurally conserved functional domains, to functionally support the observed binding and polyubiquitination of target molecules. Human Pellinos have non-redundant roles in NF-κB activation in response to pro-inflammatory stimuli such as IL-1β or the TLR4-ligand LPS (Fig. 5C). As reported previously HsPellino1 has a positive regulatory role^23^, HsPellino3 acting in an inhibitory capacity^22^ and HsPellino2 having no marked effect on NF-κB activation^8^. In human cells, SmPellino significantly (*P* < *0.001*) inhibited IL-1β- and LPS-induced activation of NF-κB to a level comparable to the inhibitory effects of HsPellino3S (Fig. 5C) and in a dose-dependent manner (Fig. 5D). Pellino proteins modulate TLR signalling via interaction with IRAK-1, TRAF-6 and TAK-1/TAB-1. To examine this functional niche for helminth proteins, we studied the effect of SmPellino overexpression on signalling induced by overexpressing various pathway members. SmPellino inhibited signalling induced by the upstream proteins MyD88, IRAK-1, TRAF-6 and TAK-1/TAB-1, but not IKKβ, which is downstream of Pellino proteins. These studies demonstrate the regulatory effects of SmPellino on ligand-induced activation of NF-κB in a human cellular system and confirm the capacity of the *S. mansoni* homolog to modulate a TLR signalling pathway.

## Discussion

The role of TLRs in pathogen recognition in innate immunity was initially elucidated using *Drosophila* as a model^3^. Many features of innate immunity are similar among vertebrates and non-vertebrates, suggesting that they have a common origin and have been conserved across millions of years^24^. Our understanding of innate immunity in higher vertebrates, including humans, has been advanced by allowing inference of the evolutionary history of immune components in non-vertebrate model organisms, facilitating the identification of functionally conserved proteins and signalling pathways. TLRs are absent from non-animal phyla but are present in most eumetazoans, with the exception of platyhelminths. Interestingly, helminth species lack the majority of genes of the canonical TLR signalling pathway and thus lack competent innate immune signal transduction. Loss of the pathway may be a result of physiological simplifications arising from the specific ancestry of the helminth lineage. Comprehensive *in silico* analysis was performed on the genome databases of all helminth species for which sequence data was available. Our results revealed that all such genes, with the exception of Pellino, have been fully lost from helminth genomes. Strict conservation of an effector protein in the absence of its niche signalling pathway is rare, therefore in this study we report on the helminth homologues of the human Pellino proteins and determine structural and functional conservation using *Schistosoma mansoni* Pellino as a molecular model. We predict that helminth Pellino proteins will provide insight into ancestral functions which may lead to the identification of novel signalling mechanisms of this important innate immune protein family.

**Figure 3:**
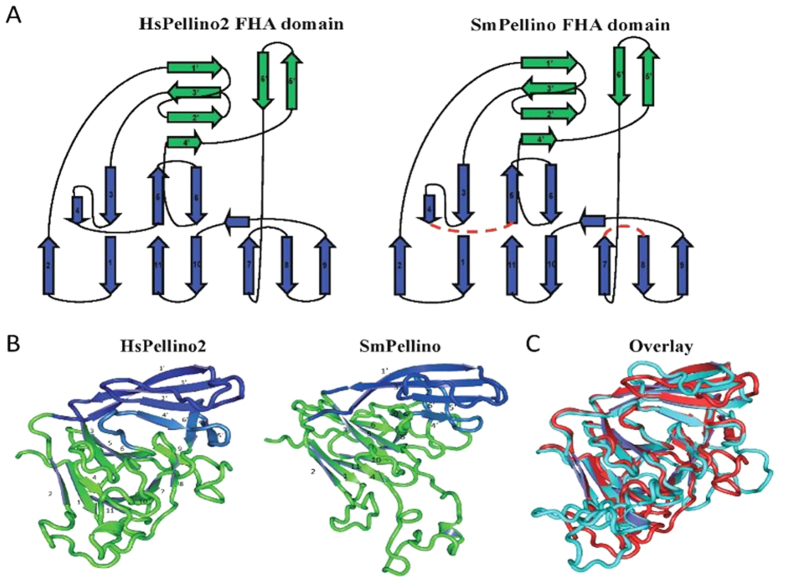
Homology modeling of helminth Pellino proteins. (**A**) Topology diagrams of Pellino2 and *S. mansoni* Pellino with beta-strands of core FHA domain in blue and of non-canonical wing in green. (**B**) FHA domain of the Pellino 2 (PDB:3EGB) crystal structure (left image), comparative model of SmPellino modeled as an FHA domain (middle image). (**C**) Pellino 2 (PDB:3EGB) crystal structure in red overlaid with the comparative model of SmPellino modeled as an FHA domain (in blue). Visualisation was performed with PyMOL (De Lano Scientific, USA).

**Figure 4:**
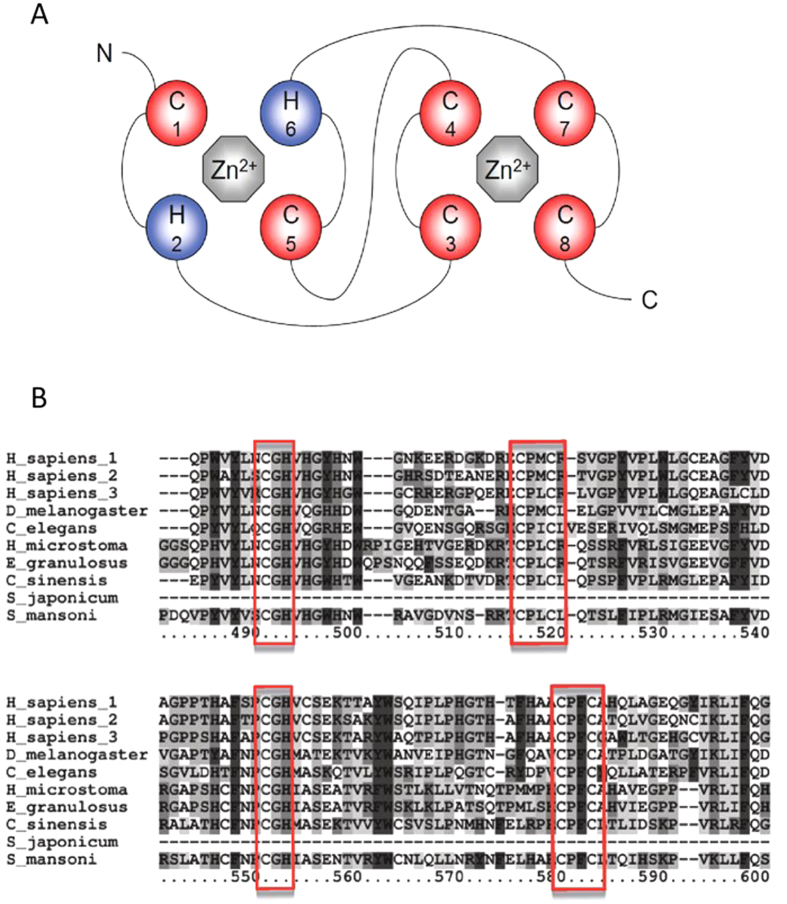
Analysis of the helmith Pellino RING-like domain by multiple sequence alignment with human, nematode and arthropod Pellino proteins. (**A**) Schematic representation of the RING-like domain of the helminth protein. The RING-like domain has an atypical CHC2CHC2 conformation. (**B**) Multiple sequence alignment of the RING-like domain containing region of human Pellino 1, 2, 3, *D*. *melanogaster* Pellino, *Brugia malayi* Pellino, *C. elegans* Pellino and various helminth Pellino proteins. The highlighted residues form the RING-like domain. The RING-like domain is conserved in all species analyzed.

**Figure 5:**
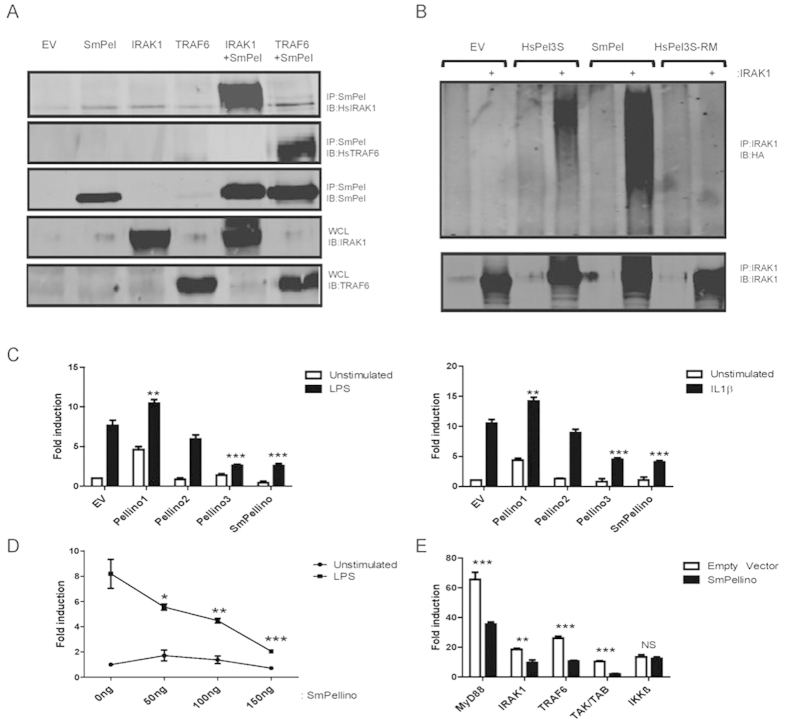
Functional characterization of SmPellino in human cells. (**A**) Transient over-expression of SmPellino-Myc with HsIRAK-1 or HsTRAF-6-Flag. SmPellino was immunoprecipitated (IP) with immunoblotting (IB) for HsIRAK-1 and HsTRAF-6 to detect protein-protein interactions. (**B**) *In vivo* ubiquitination assay was performed by co-transfecting HsIRAK-1 with or without SmPellino, HsPellino3 (HsPel3) or HsPellino3-RING-mutant (HsPel3-RM), and HA-tagged ubiquitin. IRAK-1 was IP and ubiquitin-HA detected by IB with anti-HA antibody. Expression of IRAK-1 was controlled by IB for IRAK-1 post-IP. (**C**) HEK 293-TLR4 cells were co-transfected with empty expression vector (EV) or constructs encoding HsPellino1, 2, 3S or SmPellino and NF-κB-luciferase (80 ng) and pGL3-*Renilla* luciferase (20 ng). Cells were stimulated with either IL-1β or LPS. (**D**) HEK-293 cells were transfected with increasing concentrations of EV or SmPellino in the presence/absence of LPS. (**E**) HEK-293 cells were co-transfected with either EV or SmPellino and vector for the overexpression of MyD88, IRAK-1, TRAF-6, TAK-1/TAB-1 or IKKβ to induce downstream signaling. (**C–E**) In total, 24 h post-transfection, lysates were assayed for firefly and pGL3-*Renilla* luciferase activity. Data are presented relative to cells transfected with empty vector alone and were subjected to a paired t-test. The asterisk (*) indicates that SmPellino reduces the corresponding TLR-signalling component-induced NF-kB activation with *p* < *0.05*. Results represent mean+S.E.M. of three independent experiments, each performed in triplicate.

To date, a single Pellino protein has been identified in arthropods, viruses and nematodes^21^. Helminth Pellino proteins were initially identified based on sequence identity with mammalian Pellino paralogues. Our approach to studying helminth homologues was to characterise conservation and function in relation to the well-studied human Pellino proteins. The sequence identity of helminth Pellino proteins with the human orthologues was moderate however regions of higher identity mapped to functional domains, in particular the N-terminal FHA domain. Homology modelling of helminth Pellino protein FHA domains using the human Pellino2 crystal structure as a template revealed that all helminth Pellino proteins studied had the potential to form an FHA (Fig. 3) supported further by the conservation of five signature amino acid residues in FHA domain-containing proteins that mediate direct binding to phosphorylated threonine residues on partner proteins (See Supplemental Fig. 3). Interestingly, the ‘wing’ appendages conserved in helminth pellino proteins contain sufficient serine/threonine sites for IRAK-1-mediated phosphorylation which predicts the potential of these proteins to functionally interact with target molecules. Helminth proteins also have a fully conserved RING-like domain for E3 ligase activity.

Human Pellino proteins have two functional domains, an N-terminal FHA domain that mediates binding to phosphorylated IRAK-1 and a C-terminal RING-like domain that catalyses IRAK-1 poly-ubiquitination (Fig. 1A). It should be noted, however, that helminths do not contain the IRAK family of proteins, nor do they contain TRAF-6, both well-characterized target proteins of human Pellinos^16^,^22^. We examined predicted functions of the helminth Pellino proteins using the *S. mansoni* ortholog as a molecular model. The FHA domain of *S. mansoni* is, as predicted *in silico*, functional when overexpressed in human HEK-293 cells, efficiently binding both HsIRAK-1 and HsTRAF-6. The SmPellino RING-like domain is functional and has the capacity to poly-ubiquitinate HsIRAK-1 in human cells. Such molecular conservation is supported by the capacity of SmPellino to usurp, in a dose dependent manner, human TLR/IL-1R mediated signaling *via* suppression of NF-κB. Our results indicate that signal dampening by SmPellino in human cells is similar to that of the known suppressive molecule HsPellino3^12^. Furthermore, driving the signalling pathway by overexpressing effector proteins traces the inhibitory function of SmPellino to the interaction with IRAK-1 and TRAF-6. SmPellino is, therefore, a conserved signaling molecule in both structure and function, with the ability to bind, post-translationally modify and mediate human TLR signalling when over-expressed in human cells. Using *S. mansoni* as a model to study helminth Pellino protein function suggests that other helminth Pellino proteins, which have similar levels of homology in their sequence and structure, are also functional. In the absence of IRAK-1 and TRAF-6 effector proteins, we suggest that helminth Pellinos modulate alternate target proteins that may be present in the helminth via these conserved intrinsic mechanisms.

In helminths, phosphorylation and ubiquitination play important roles in maintaining homeostasis and regulating complex cellular adaptations^25^,^26^. Helminths process extracellular signals from the environment *via* specialized sensory receptors, inducing biological change through non-linear intracellular signal transduction pathways^25^. Phosphorylation, and indeed de-phosphorylation, of serine and threonine residues specifically regulates the activity status of effector and adaptor proteins to facilitate the integration of signalling networks. Furthermore, there are a number of ubiquitination-related proteins expressed in helminths, including ubiquitin-conjugating enzymes (Ub-E2), small-ubiquitin related modifier (SUMO), the SUMO-pathway homologues SmT3B and SmT3C, and a ring-box protein (SmRbx)^26^,^27^,^28^. These proteins are functionally homologous to mammalian proteins with differential expression profiles throughout the life cycle of helminths. The processes by which mammalian Pellino proteins are regulated and their regulatory targets are, therefore, potentially functional in helminths.

Using string-db, the first-in-class software for recording evidence of protein interaction networks^29^, we have analysed alternative pathways in which mammalian Pellino proteins have been implicated. Interestingly, but unsurprisingly, Pellino interacts with ubiquitin-C (UBC) proteins in mammals^30^. UBC is also encoded in helminth genomes, known from experimental evidence at the transcriptome level in *S. japonicum*^31^. This supports the hypothesis for E3 ligase activity of helminth Pellino proteins *in vivo*. Pellino proteins have also been shown to regulate responses to viruses via TLR signalling pathways^32^, however, homologues of this pathway are also absent from platyhelminths. Furthermore, human Pellino3 (the protein to which helminth Pellino has most functional compatibility) regulates JNK, ERK and p38 MAPK activity in response to TLR signalling^33^. Indeed, the stress-activated JNK protein is present in *S. mansoni, S. japonicum, H. microstoma* and *E. granulosus*^34^. An ERK and p38 MAPK homologue is also present in *S. mansoni* and *S. japonicum*^35^, with no evidence for expression in other platyhelminths to date. This suggests a potential mechanism for modulation of helminth signalling via alternate MAPK pathways to NF-κB. The role of helminth Pellino proteins in the parasite is of considerable interest for understanding ancestral signalling mechanisms and also for helminth biology, including the development of new drugs. The use of RNA interference (RNAi) on schistosome worms^36^ would be informative to determine the effects of knockdown of the SmPellino gene on worm biology as well as functions of the protein in worm immunity.

In the helminth parasite, the retention of the innate signaling intermediary protein, Pellino, while both the upstream Toll receptors that initiate the signaling cascade and downstream activating gene NF-κB have been lost, highlights the biological function of helminth Pellino proteins within the parasite as an important question to be addressed as it may reveal novel functions for human Pellino proteins. In conclusion, this study provides for the first time a detailed characterization of a helminth homolog of the Pellino family of signaling proteins and identifies a potential evolutionary signal between intracellular proteins of host and parasite.

## Materials and Methods

### *Schistosoma mansoni* life cycle maintenance and infection of mice

Adult *S. mansoni worms* were recovered by portal perfusion of infected mice 49 days post-infection. Worms were immediately transferred to MEM containing Earle’s salts and Sodium bicarbonate. All animal experiments were performed in compliance with Irish Department of Health and Children regulations and approved by the Trinity College Dublin’s BioResources ethical review board.

### Amplification and cloning of SmPellino

Total RNA was extracted from adult worms using Trizol reagent (Sigma) and treated with DNAse I (Sigma). Total RNA was used for reverse transcription with SuperScript III First Strand Synthesis SuperMix (Invitrogen), using 50 ng of Oligo(dT) according to manufacturer’s instructions. The 5′end and 3′end sequences were obtained by rapid amplification of cDNA ends (RACE, Invitrogen). PCR products were cloned into pCR®4-TOPO (Invitrogen), transformed into TOP10 competent *E. coli* cells and propagated for gene sequencing. *S. mansoni* Pellino codon sequence was optimized for expression in mammalian cells and chemically synthesised (GeneArt) before subcloning into the pcDNA3.1+ mammalian expression vector, via pDONR221 vector (Invitrogen). Human IRAK-1 and TRAF-6 in pcDNA3.1+ were used created using the same protocol.

### HEK 293 cell culture and transfection

The parental human embryonic kidney (HEK) 293 cells and TLR4-expressing HEK 293 cells were maintained in DMEM supplemented with 10% FBS 100 U/mL penicillin + streptomycin, and 2 mM L-glutamine. G418 (Geneticin, Sigma, 500 μg/mL) was used to select for the stably transfected TLR cell lines. Seeded cells at ~80% confluency were transfected using Lipofectamine 2000 (Invitrogen) using a DNA to transfection agent ratio of 1:3.

### Protein Immunoprecipitation and Western blot analysis

Twenty-four hours post-transfection, cells were lysed in 50 mM Tris-HCl (pH 7.5) containing 150 mM NaCl, 0.5% v/v Igepal, 50 mM NaF, 1 mM Na3VO4, 1 mM dithiothreitol, 1 mM phenylmethylsulfonyl fluoride, protease inhibitor mixture (25 mg/mL leupeptin, 25 mg/mL aprotinin, 1 mM benzamidine and 10 mg/mL trypsin inhibitor). An aliquot of supernatant was retained for Western blot analysis and the remainder was subjected to immunoprecipitation (IP) using monoclonal antibodies for specific affinity epitopes – anti-myc antibody (Cell signaling, 9B11) and anti-flag antibody (cell signaling, 2368). Lysates were pre-cleared by addition of IgG antibody and re-suspended Protein A/G-agarose. IP with the appropriate antibody was performed overnight at 4 °C. Antibody–protein complexes were pelleted after addition of Protein A/G-agarose. Samples were boiled in reducing sample buffer and immunoprecipitates subjected to SDS-PAGE and Western blot analysis

### NF-κB Luciferase reporter assay

The PathDetect NF-κB cis-reporting system (Stratgene) was used, according to the manufacturer’s recommendations, to measure activation of the NF-κB pathway. Briefly, HEK 293 and HEK 293-TLR4 cells were then transfected with the NF-κB-regulated firefly luciferase reporter plasmid pNF-κB-Luc, constitutively expressed *Renilla*-luciferase reporter construct (pGL3-*Renilla*) and with or without human Pellino1, 2, 3S and SmPellino expression constructs. Twenty-four hours post-transfection, the medium was removed from the cells and lysed with reporter lysis buffer (Promega). Firefly luciferase activity was assayed using firefly luciferase substrate (Promega), while *Renilla*-luciferase activity was assayed using coelenterazine (Insight Biotech.) in PBS. Luminescence was assayed using a Glomax microplate luminometer (Promega).

### Protein Structure Predictions

The crystal structure of the human Pellino2 proteins (PDBs: 3EGA and 3EGB^18^) has previously been used as a template for building Pellino homology models^21^. The sequence identity to the Pellino2 crystal structures was quantified using the pairwise alignment Pro-align in MOE (MOE 2008 http://www.chemcomp.com). Accelrys Discovery Studio 3.5 was used to prepare the protein structures. The protein sequences were aligned to the template of the known human Pellino2 structure (PDB: 3EGA, 3EGB) using Discovery Studio 3.5 and 1,000 protein structures were built for each alignment. The Modeller software implemented comparative protein structure modeling, by satisfying spatial restraints^37^,^38^. The alignment is used to construct a set of geometrical criteria that are converted into probability density functions (PDFs) for each restraint. A global optimization procedure refines the positions of all heavy atoms in the protein. The best model was selected using a combination of the Modeller discrete optimized protein energy (DOPE) score and a selection of protein assessment tools.

Profiles 3D (Accelrys Discovery Studio 3.5)^39^, PROCHECK^40^ and ERRAT^41^ were used to check the generated models and count the number of non-bonded interactions between atoms (CC, CN, CO, NN, NO, and OO) within a cutoff distance of 3.5 Å to yields an overall quality factor for each structure, which is expressed as the % of protein for which the calculated error value falls below a 95% rejection limit. The final model selected yielded the overall best performance across the validation tools.

### Molecular dynamics simulations

The protein structure predictions were embedded in a solvated box. MD simulations were performed using the NAMD 2.10 simulation package^42^. The CHARMM22 force field^43^,^44^ was used for proteins and water molecules were described using TIP3P^45^. All systems were simulated at 310 K. Temperature and pressure were held constant with Langevin dynamics and the Nose-Hoover Langevin piston. Particle-mesh Ewald was used to calculate electrostatic interactions and a 12 Å cut-off for van der Waals interactions was used. Briefly, positional harmonic restraints were used on the protein and then protein backbone. The restraints were reduced at each subsequent equilibrium simulation. The first two simulations used the NVT (constant volume and temperature) ensemble. A timestep of 1 fs was used for the restrained equilibrium simulations, which were 0.1 ns each. Equilibration without restraints was performed for 1 ns. Production runs began after the systems were equilibrated and used an NPT (constant pressure and temperature) ensemble and a 2 fs timestep. Harmonic restraints were not used in the production runs. Production runs were for 50 ns.

### Data analysis

Visual Molecular Dynamics 1.9.1 (VMD)^46^ was used to visualize the trajectories and to perform the all-to-all RMSD calculations and the salt bridge analysis. The timeline plugin was used for viewing temporally changing per-residue attributes of the molecular structures.

### Bioinformatic analysis of protein primary structures

Human and *Drosophila* Pellino protein sequences were used as queries to perform a BLAST analyses (http://blast.ncbi.nlm.nih.gov/Blast.cgi/) of known translated nucleotide sequences in helminth genomic DNA (v5.2, released 2/5/2014) sequence downloaded from Welcome Trust Sanger Institute ftp website (http://www.sanger.ac.uk/resources/downloads/helminths/). Protein signatures were identified using Interproscan sequence search for assignments of protein signatures (http://www.ebi.ac.uk/Tools/pfa/iprscan/). Alignment of multiple sequences was performed using MUSCLE software (http://www.ebi.ac.uk/Tools/msa/).

### Phylogenetic analysis

Phylogenetic analyses were performed upon a subset of all organisms containing Pellino homologues. The subset was chosen using the OMA browser database and supplemented with putative Pellino protein sequences manually identified by BLAST analysis. Model fitting analysis was performed using Prottest^47^. Phylogenetic construction was using RaxML 7^48^ under the LG model with a gamma correction. 100 bootstraps were performed with the RaxML fast-bootstrapping method.

## Additional Information

**How to cite this article**: Cluxton, C. D. *et al.* Functional conservation of an ancestral Pellino protein in helminth species. *Sci. Rep.* **5**, 11687; doi: 10.1038/srep11687 (2015).

## Supplementary Material

Supplementary Information

## Figures and Tables

**Table 1:** Comparative analysis of the canonical TLR signalling pathway from humans and selected insects, nematodes and helminth species.

**Drosophila melanogaster**	**TLR (AGB96375.1)**	**MyD88 (AAF58953.1)**	**IRAK (AAF56686.1)**	**TRAF6 (AAF46338.1)**	**Pellino (ABW08739.1)**	**IKK (AAF53911.2)**	**IκB (AAA85908.1)**	**NFκB (AAA28465.1)**
***Homo sapiens***	***8e-31***	***8e-20***	***3e-41***	***2e-42***	***1e-177***	***7e-156***	***2e-34***	***1e-57***
***Caenorhabditis elegans***	***6e-26***	*N.D*	*N.D*	*N.D*	***6e-127***	*N.D*	*N.D*	*N.D*
***Brugia malayi***	*N.D*	*N.D*	*N.D*	*N.D*	***2e-132***	*N.D*	*N.D*	*N.D*
***Dugesia japonicum***	*N.D*	*N.D*	*N.D*	4e-13*	*N.D*	*N.D*	*N.D*	*N.D*
***Schmidtea mediterranea***	*N.D*	*N.D*	*N.D*	5e-06*	*N.D*	*N.D*	*N.D*	*N.D*
***Clonorchis sinensis***	*N.D*	*N.D*	*N.D*	7e-11*	***2e-103***	*N.D*	*N.D*	*N.D*
***Echinococcus granulosus***	*N.D*	*N.D*	*N.D*	8e-06*	***2e-83***	*N.D*	*N.D*	*N.D*
***Hymenolepis microstoma***	*N.D*	*N.D*	*N.D*	1e-08*	***1e-89***	*N.D*	*N.D*	*N.D*
***Schistosoma japonicum***	*N.D*	*N.D*	*N.D*	6e-07*	***5e-50***	*N.D*	*N.D*	*N.D*
***Schistosoma mansoni***	*N.D*	*N.D*	*N.D*	5e-06*	***9e-70***	*N.D*	*N.D*	*N.D*

BLAST expectation scores are reported to quantify predicted homology with known signaling proteins.

^*^Positive results are an artefact of the high sequence similarity with TRAF2, an orthologue previously identified in helminths.
