# Supplementary material for: Functional conservation of an ancestral Pellino protein in helminth species
Source: Sci Rep. 2015 Jun 29;5:11687. doi: 10.1038/srep11687 (PMC4484250; doi:10.1038/srep11687)
Supplement: Supplementary Information [file srep11687-s1.pdf]

Functional conservation of an ancestral Pellino protein in helminth species

Christopher D. Cluxton[1], Brian E. Caffrey[2], Gemma K. Kinsella[3], Paul N. Moynagh[3], Mario A. Fares[2,4] & Padraic G. Fallon[1,5]*.

Supplemental table 1: Sequence identity between helminth and human Pellino proteins

SeqA	*Name*	SeqB	*Name*	Score
1	*H_microstoma/1-541*	6	*H_sapiens_1/1-418*	**43.78**
1	*H_microstoma/1-541*	7	*H_sapiens_2/1-420*	**41.67**
1	*H_microstoma/1-541*	8	*H_sapiens_3/1-469*	**38.38**
2	*E_granulosus/1-562*	6	*H_sapiens_1/1-418*	**41.63**
2	*E_granulosus/1-562*	7	*H_sapiens_2/1-420*	**39.76**
2	*E_granulosus/1-562*	8	*H_sapiens_3/1-469*	**37.1**
3	*C_sinensis/1-574*	6	*H_sapiens_1/1-418*	**46.41**
3	*C_sinensis/1-574*	7	*H_sapiens_2/1-420*	**44.76**
3	*C_sinensis/1-574*	8	*H_sapiens_3/1-469*	**39.66**
4	*S_japonicum/1-252*	6	*H_sapiens_1/1-418*	**44.84**
4	*S_japonicum/1-252*	7	*H_sapiens_2/1-420*	**44.84**
4	*S_japonicum/1-252*	8	*H_sapiens_3/1-469*	**44.84**
5	*S_mansoni/1-606*	6	*H_sapiens_1/1-418*	**43.3**
5	*S_mansoni/1-606*	7	*H_sapiens_2/1-420*	**43.57**
5	*S_mansoni/1-606*	8	*H_sapiens_3/1-469*	**38.38**

Supplemental table 2: Protein structure validation of the template, HsPellino2 and resultant Helminth Pellino homology models.

	**Procheck** (core, allowed, generously allowed, disallowed)	**Verify_3D***	**Errat**
**3EGA**	85.6% core; 12.3% allowed; 1.6% generously allowed; 0.5% disallowed	95.81%	89.062
**3EGA HM**	84.4% core 11.8% allowed; 2.8% generously allowed; 0.9% disallowed	79.18%	85.593
E_granulosus	71.6% core; 25.3% allowed; 2.6% generously allowed; 0.4% disallowed	82.67%	72.180
H_microstoma	76.8% core; 19.9% allowed 2.5% generously allowed; 0.8% disallowed	68.33%	84.677
S_japonicum	74.5% core; 21.4% allowed; 2.7% generously allowed; 1.4% disallowed	51.79%	77.686
S. mansoni	74.2% core; 21.2% allowed; 2.9% generously allowed 1.7% disallowed	72.22%	77.606

*Percentage of the residues with an averaged Verify_3D 1D score > 0.2

Supplemental table 3: Structural Analysis of representative snapshots post molecular dynamics (MD).

	Procheck (core, allowed, generously allowed, disallowed)	Verify_3D*	Errat
**3EGA HM**	88.1% core; 9.5% allowed; 1.9% generously allowed; 0.5% disallowed	88.70%	89.894
E_granulosus	83.3% core; 15.4% allowed; 0.9% generously allowed 0.4% disallowed	83.39%	85.075
H_microstoma	86.7% core; 11.2% allowed; 1.7% generously allowed 0.4% disallowed	71.79%	90.449
S_japonicum	85.8% core; 13.2% allowed; 0.9% generously allowed; 0.0% disallowed	74.80%	85.030
S. mansoni	87.9% core 11.7% allowed; 0.4% generously allowed; 0.0% disallowed	74.34%	86.486

*Percentage of the residues with an averaged Verify_3D 1D score > 0.2

Supplemental table 4: Root mean square deviation (RMSD) of final snapshot of each simulation compared with the template HsPellino2 structure.

	HsPellino2<br><br>**Res 15-257**	***E_granulosus***<br><br>**Res 26-300**	***H_microstoma***<br><br>**Res 26-304**	***S_japonicum***<br><br>**Res 3-251**	***S. mansoni***<br><br>**Res 3-270**
**RMSD (Angstrom)**	1.090	2.045	1.133	2.191	1.435

Supplemental table 5: Comparison of the secondary structure regions observed in the HsPellino2 crystal structure (PDB: 3EGA) and the modelled Helminth Pellino structures.

β- Strands	3EGA	*HsPellino2*	*E_granulosus*	*H_microstoma*	*S_japonicum*	*S. mansoni*
		**Res 15-257**	**Res 26-300**	**Res 26-304**	**Res 3-251**	**Res 3-270**
**1**	18-24	18-25	29-36	29-36	6-11	6-12
**2**	42-46	42-47	54-59	54-58	30-33	31-36
**1'**	53-63	54-63	66-74	66-75	40-50	42-53
**2'**	77-82	78-82	90-93	89-93	65-69	67-72
**3'**	87-96	87-95	100-107	99-107	74-84	76-85
**3**	99-106	99-105	111-117	111-117	86-92	89-94
**4**	114-115	114-115	126-127	126-127	101-103	103-104
**5**	141-146	141-146	181-186	183-190	147-152	148-153
**6**	153-157	153-157	193-196	197-201	159-163	160-164
**4'**	165-169	165-168	205-209	209-212	171-175	172-176
**5'**	174-177	174-177	214-216	218-220	179-183	181-183
**6'**	181-184	182-184	221-223	225-227	187-190	189-190
**7**	190-194	190-193	231-233	234-236	196-197	197-200
**8**	208-211	209-211	250-255	256-258	222-224	223-227
**9**	217-219	217-218	260-262	264-265	230-232	233-235
**10**	245-248	245-248	288-290	291-294	-	258-260
**11**	252-257	252-256	295-299	299-303	-	265-269

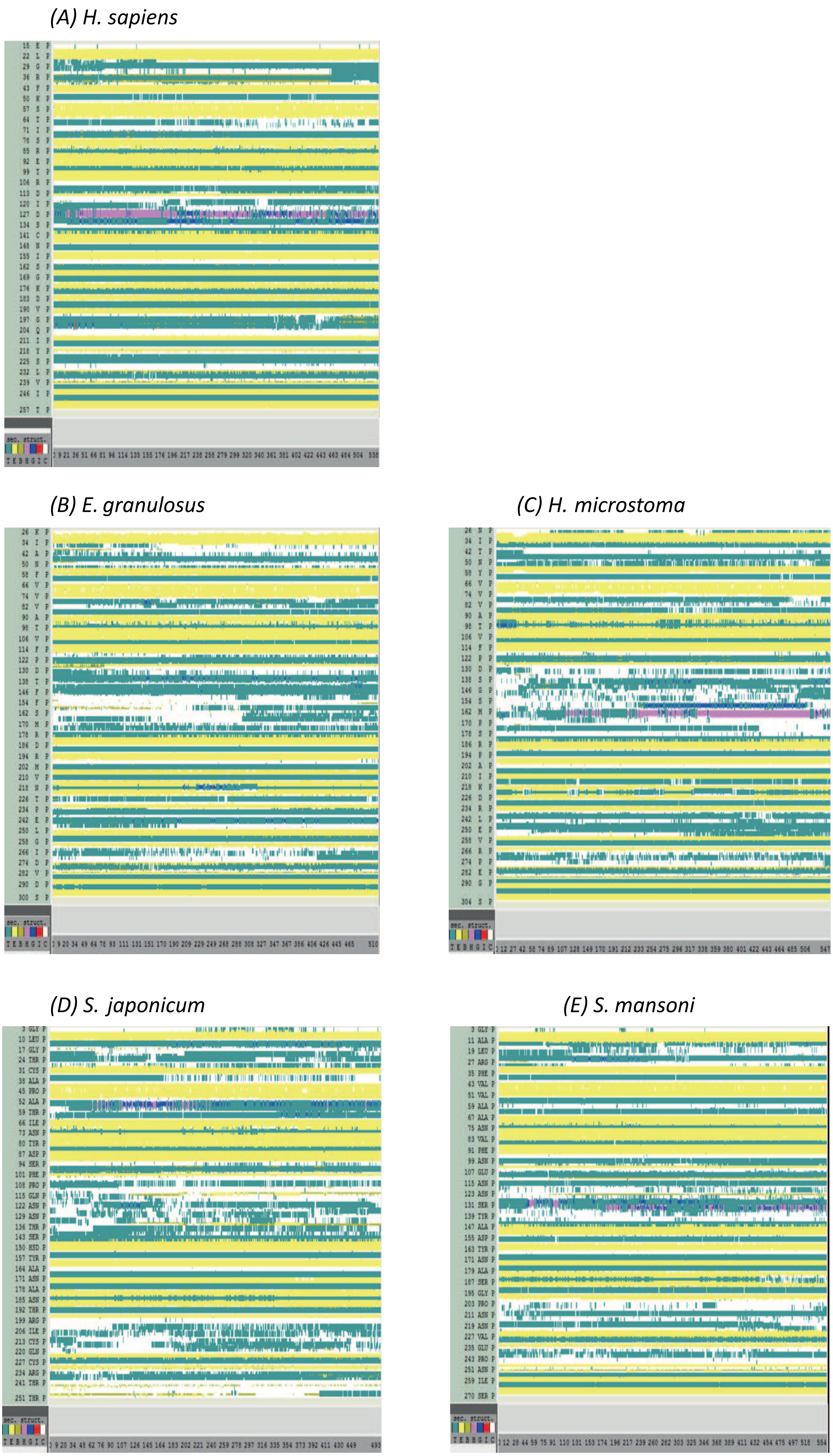

(A) H. sapiens
(B) E. granulosus
(C) H. microstoma
(D) S. japonicum
(E) S. mansoni

Figure S1

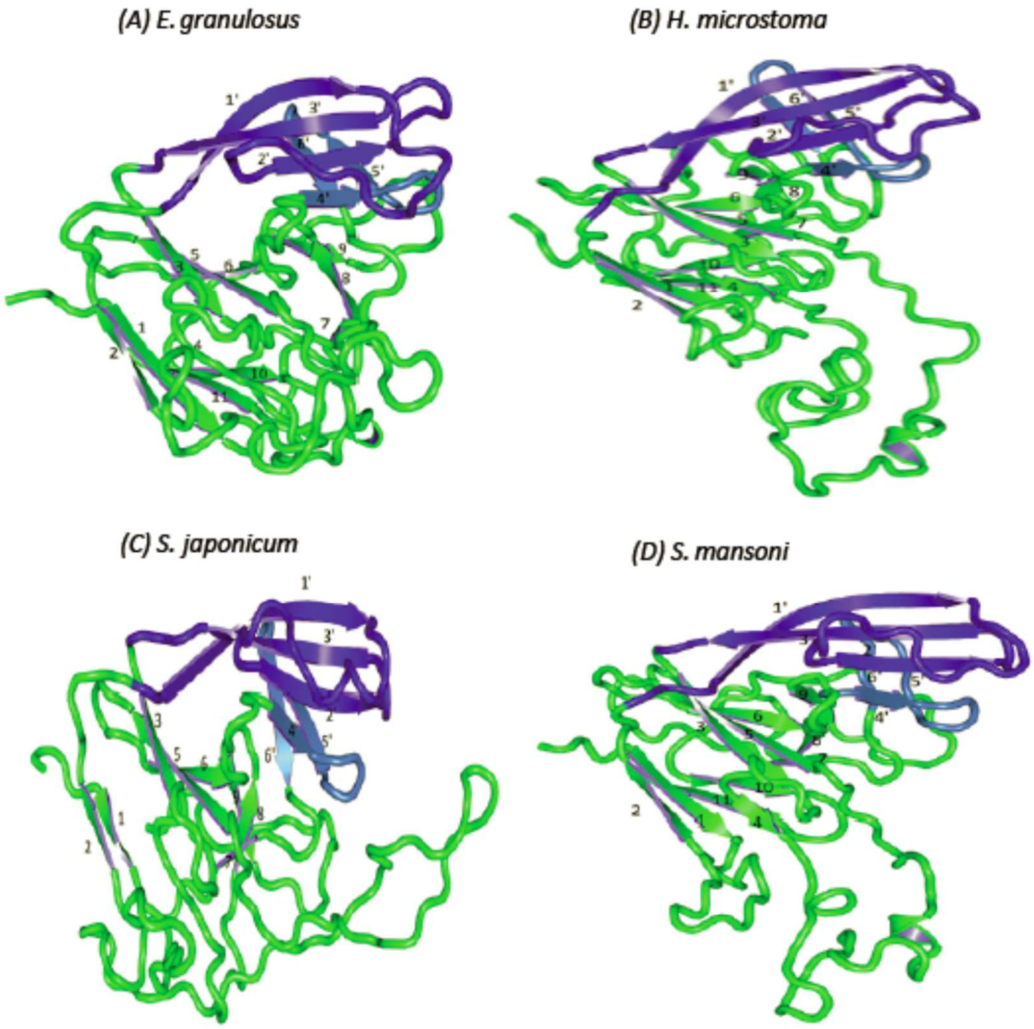

Figure S2

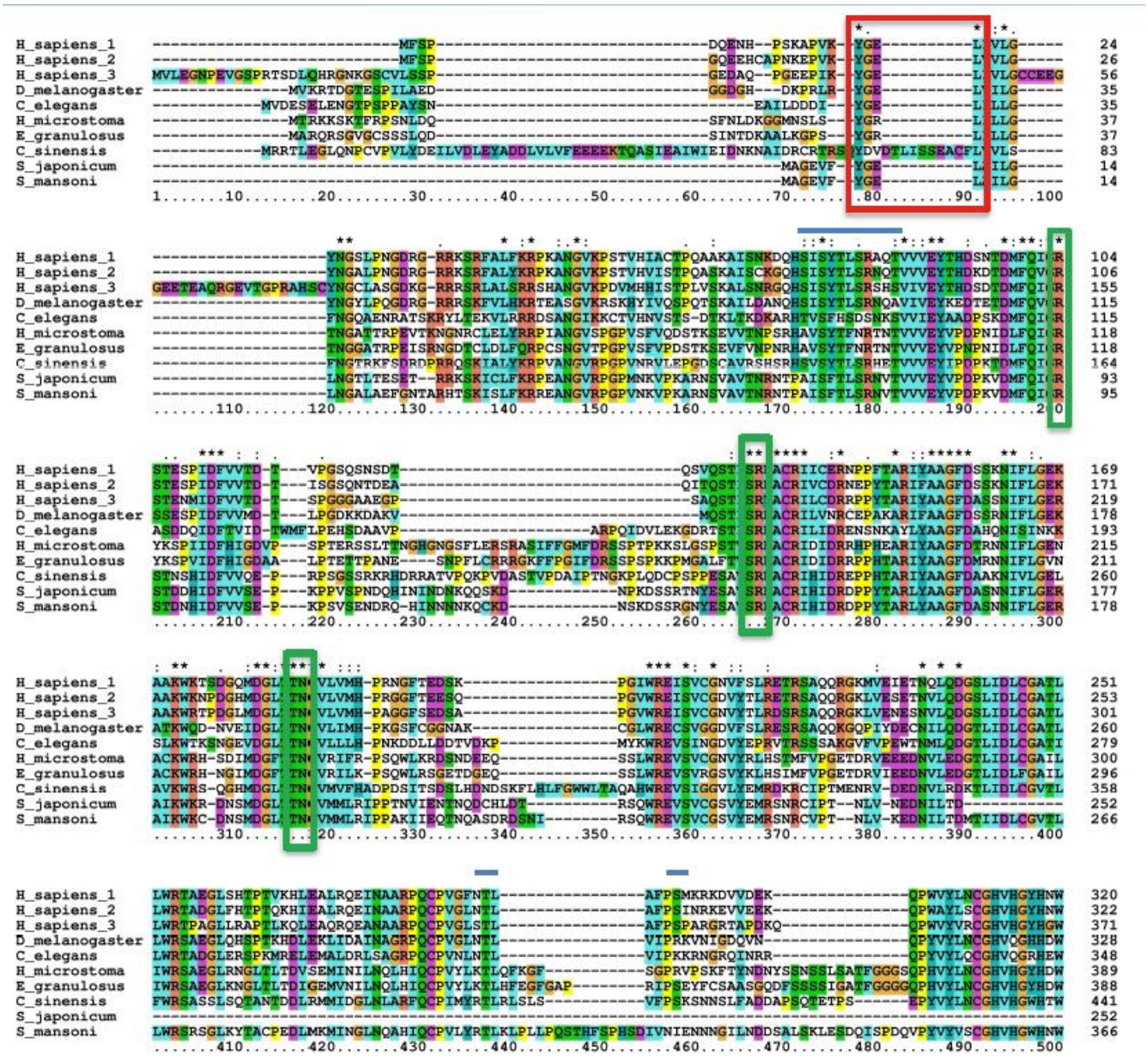

Figure S3

Supplementary Figure legends

Supplementary table 1: Sequence homology between helminth and human Pellino proteins.

Supplementary table 2: Analysis of the template, HsPellino2 and resultant helminth Pellino homology models with the protein structure validation tools, Procheck, Errat and Verify 3D. 3EGA_HM is the completed HsPellino2 structure with missing loops modelled.

Supplementary table 3: Structural Analysis of representative snapshots post molecular dynamics (MD).

Supplementary table 4: Root mean square deviation (RMSD) of final snapshot of each simulation compared with the template HsPellino2 structure.

Supplementary table 5: Comparison of the secondary structure regions observed in the HsPellino2 crystal structure (PDB: 3EGA) and the modelled helminth Pellino structures.

Supplementary figure (S) 1: Secondary Structure timeline analysis of homology models as computed by the timeline plugin contained in VMD. Images represent results for (A) *Homo sapiens* (B) *Echinococcus granulosus* (C) *Hymenolepis microstoma* (D) *Schistosoma japonicum* and (E) *Schistosoma mansoni*. In the graphic, the β-sheet components turn (T) and extended conformation (E) are represented in teal and yellow respectively, isolated bridges are in dark yellow, degrees of helix are in pink (α-helix), blue (3-10 helix) and red (π-helix), random coils are in white.

Supplementary figure (S) 2: Representative snapshots of *E. granulosus* (A), *H. microstoma* (B), *S. japonicum* (C), *S. mansoni* (D) Pellino after 50ns MD simulation. β strands of the core FHA domain are in blue and of the non-canonical wing are in green. Image generated using Pymol.

Supplementary figure (S) 3: Helminth Pellino proteins contain functional residues found in human Pellino proteins. The YGEL sequence, that has proven important for IRAK-1 binding and Pellino signaling to p38 MAPK, is partially conserved and marked with a red box. Five residues essential for Pellino protein binding to phosphothreonine residues on target proteins are conserved and are marked with green boxes. Human Pellino protein phosphorylation is mediated via a number of serine and threonine residues. Smith et al (2009) has shown that any of Ser-76, Thr-86, Thr-288, or Ser-293 or a combination of Ser-78, Thr-80, and Ser-82 residues is sufficient to activate Pellino proteins. SmPellino retains Ser-78, Thr-80, Ser-82, Thr-86, and Thr-288, thus maintaining activating potential, similar to that of human Pellino proteins and are highlighted with a purple bar.
